# Antitumor responses in gastric cancer by targeting B7H3 via chimeric antigen receptor T cells

**DOI:** 10.1186/s12935-022-02471-8

**Published:** 2022-01-31

**Authors:** Fengqiang Sun, Xiaomei Yu, Ruixue Ju, Zhanzhao Wang, Yuhui Wang

**Affiliations:** 1 Department of Clinical Laboratory, Weifang People’s Hospital, Weifang, 261000 Shandong China; 2 Department of Obstetrics, Weifang People’s Hospital, Weifang, 261000 Shandong China

**Keywords:** Gastric cancer, B7H3, CAR-T cell, Cancer stem cell, Clinical application

## Abstract

**Background:**

Gastric cancer (GC) has a poor prognosis and limited therapeutic options. As a new promising cancer therapeutic approach, chimeric antigen receptor (CAR)-T cells represent a potential GC treatment. We investigated the antitumor activity of CAR-T cells target-B7H3 in GC.

**Methods:**

In our study, expression of B7H3 was examined in GC tissues and explored the tumoricidal potential of B7H3-targeting CAR-T cells in GC. B7H3-directed CAR-T cells with a humanized antigen-recognizing domain was generated. The anti-tumor effects of this CAR-T cell were finally investigated in vitro and in vivo.

**Results:**

Our results show that B7H3-directed CAR-T cells efficiently killed GC tumor cells. In addition, we found that B7H3 is correlated with tumor cell stemness, and anti-B7H3 CAR-T can simultaneously target stem cell-like GC cells to improve the treatment outcome.

**Conclusions:**

Our study indicates that B7H3 is an attractive target for GC therapy, and B7H3 has high potential for clinical application.

**Supplementary Information:**

The online version contains supplementary material available at 10.1186/s12935-022-02471-8.

## Background

Gastric cancer is a death-dealing disorder despite its declining incidence [1]. Moreover, GC is often identified in the advanced stage also corresponding with a poor prognosis for patients [2]. Although traditional treatment regimens have reduced the incidence and mortality rates of GC, the overall 5-year survival rate remains low at approximately 25% [3]. With deepened understanding of the molecular biological characteristics of gastric cancer, new gastric cancer treatment strategies were examined, and monoclonal antibody-targeted drug therapy and immunotherapy brought hope to patients with gastric cancer [4]. Tumor immunotherapy is a new kind therapy that used immunological principles and methods to activate a patient's own immune system and enhance the patient's anti-tumor immune response, which inhibits tumor growth [5].

As a therapeutic strategy for malignant diseases, chimeric antigen receptor (CAR) T cells have therapeutic potential in antitumor treatment [6]. Because of the specific expression of CD19 in malignant cells, CD19-specific CAR-T cells have significant efficacy against hematologic malignancies [7]. As a result, more than five CAR-T cell products have been used clinically in the treatment of hematologic malignancies but not yet for solid tumors [8]. In solid tumors, the function of CAR-T cells is restricted to antigen specificity [9]. Therefore, it is imperative to seek for new potent antigen.

B7H3 (also called CD276) is an immune checkpoint member and a costimulatory/coinhibitory immunoregulatory protein of the B7 family [10]. The human B7H3 gene is located on chromosome 15. Transmembrane B7H3 is a type I transmembrane protein that contains 316 amino acids [11]. It has been reported that B7-H3 preferentially overexpressed in various tumors but low expression in normal tissues [12]. As a result, it has been explored as the target of CAR-T cells against solid tumors [13], including ovarian cancer, esophagus squamous cell carcinoma, pancreatic adenocarcinoma, and brain tumors [14–17]. B7H3 targeted treatment has shown preliminary benefits for patients with tumors and is being evaluated by numerous ongoing clinical trials. However, it remains unclear whether B7-H3 is extensively overexpressed in GC and if it would be suitable for CAR-T cell therapy in patients with GC. In this study, we explored B7H3 expression in GC tissues and peritumor tissues, and detected the anti-tumor effects of B7H3-spetific CAR-T cells.

## Materials and methods

### Cell lines

Human GC cell lines (AGS, MKN45, NCI-N87, MGC803) and the human gastric epithelial cell line (GES-1) were purchased from the Cell Bank of the Chinese Academy of Sciences (Shanghai, China). 293T cells were maintained in our laboratory. All cell lines were cultured in Dulbecco’s modified Eagle’s medium (DMEM) (Gibco, USA) supplemented with 10% (v/v) foetal bovine serum (FBS) (Invitrogen) in 5% CO_2_ atmosphere at 37 °C. Lentiviral transduction were used to establishing firefly luciferase (FFluc)-expressing cells.

### Generation of CAR-T cells

Human peripheral blood CD3^+^ T lymphocytes were isolated using Human T-cell Isolation kit (Miltenyi, DEU). CD3/CD28 T-cell activation Dynabeads (Gibco, USA) was used for T cell stimulation in the presence of IL-2 (PeproTech, USA). The lentiviral plasmid containing sequences of 8H9S3.3-ScFv (or FMC63-ScFv), human CD8 transmembrane sequence, cytoplasmic domains of human 41BB and human CD3 complex ζ chain were synthesized by Sangon Bitech (Shanghai, China). The backbone for lentiviral plasmid (pCDH-CMV-MCS-EF1-GFP) and packaging plasmids (PMD2.G, PSPAX2) were purchased from addgene. 293T cells and Lip3000 were used for lentiviruses production. CD3^+^ T lymphocytes were infected with lentivirus and infection efficiency was verified according to GFP expression.

### RT-PCR

Paraffin‐embedded tumor and normal adjacent tissues of GC patients were collected from pathology department of Weifang People’s Hospital. Total-RNA of GC tumors and cell lines were extracted by the RNeasy Mini Kit according to the manufacturer’s instruction. And then the cDNA was synthesized using reverse transcription kits (TaKaRa). The sequences of primers are shown in Additional file 2: Table S1.

### Immunohistochemistry analysis

Paraffin‐embedded tumor and normal adjacent tissues of GC patients were collected from pathology department of Weifang People’s Hospital. Clinical characteristics in GC patients are shown in Additional file 3: Table S2. Immunohistochemical staining was performed with antibodies against human B7H3 (Abcam, UK). Two experienced pathologists assessed all staining results independently. Each specimen received a score according to the intensity of stained cells (0 = none, 1 = low, 2 = moderate, and 3 = high) and the extent of stained cells (0% = 0, 1–24% = 1, 25–49% = 2, 50–74% = 3, and 75–100% = 4), and the score was calculated by multiplying the intensity score by the extent of stained cells.

### TCGA data

Gene expression in Gastric cancer was screened in The Cancer Genome Atlas (TCGA) database (http://www.canc-ergen-ome.nih.gov), and Spearman’s correlation analysis was performed.

### Flow cytometry

Expression of cell surface antigen was detected by flow cytometry. B7H3 was stained with Anti-Human B7H3 (Abcam, UK), CCR7 was stained with Anti-Human CCR7 (Abcam, UK), CD45RO was stained with Anti-Human CD45RO (Abcam, UK), CD44 was stained with Anti-Human CD44 (Abcam, UK), CD133 was stained with Anti-Human CD133 (Abcam, UK).

### Cytotoxicity assay

Lactate dehydrogenase assay (LDH assay) (Abcam, UK) was used to detected the specific killing activity of CAR-T cells toward target cells. The manufacturer’s instructions were followed. Target cells were co-cultured with CAR-T cells at various E:T ratios for 24 h, supernatants were collected and incubated with Cytotox 96 regent for 30 min. Finally, stop solution were added to each well followed by quantification of absorbance at 490 nm.

### Proliferation assay

CCK8 assay (MCE, USA) was used to detected the proliferation of T cells. Around 1000 T cells were seeded in each well of 96-well plate, and 10 μL CCK8 reagent was added to the culture medium 4 h before analysis. The OD value of the culture was detected under 450 nm.

### ELISA assay

The supernatants of target cells co-cultured with CAR-T cells were probed for effector cytokines (IL-2, IFN-γ, TNF-α) using ELISA kits (Biolegend, USA) according to the manufacturer’s instructions.

### Sphere formation assay

GC cells (5 × 10^3^) were seeded onto 24-well ultra-low attachment plates using DMEM/F12 medium (containing 4 μg/mL heparin, 20 ng/mL fibroblast growth factor, 20 ng/mL EGF, B27(1:50)). Spheres were dissociated after 7 days, and sedimented cells were used for subsequent assays.

### Animal experiments

NOD-SCID mice (female, 6–8 weeks old) were obtained from Beijing Vital River Laboratory Animal Technology. All mice were housed in in a specific pathogen free (SPF) environment. Single-cell suspensions of tumor cells was injected subcutaneously. The CAR-T cells were injected intravenously. BLI was used to monitoring the tumor growth in different groups. Animal experimental protocols were compliant with the requirements of the Ethical Committee.

### Survival analysis

Kaplan–Meier survival analysis was used to analyse the relationship between overall survival and B7H3 expression in GC patients, and p < 0.05 was considered significant. The expression levels of the samples were grouped as high and low based on the cut‐off point.

### Statistical analysis

GraphPad Prism 8 was used for statistical analyses. Student’s t test, one- or two-way ANOVA analysis with a Tukey’s post hoc test was performed to evaluate the statistical significance. The level of significance was set to p < 0.05. Survival curves were prepared via the product-limit method of Kaplan and Meier, and comparisons were analyzed with using the log-rank test.

## Results

### B7H3 is highly expressed on GC

**Fig. 1:**
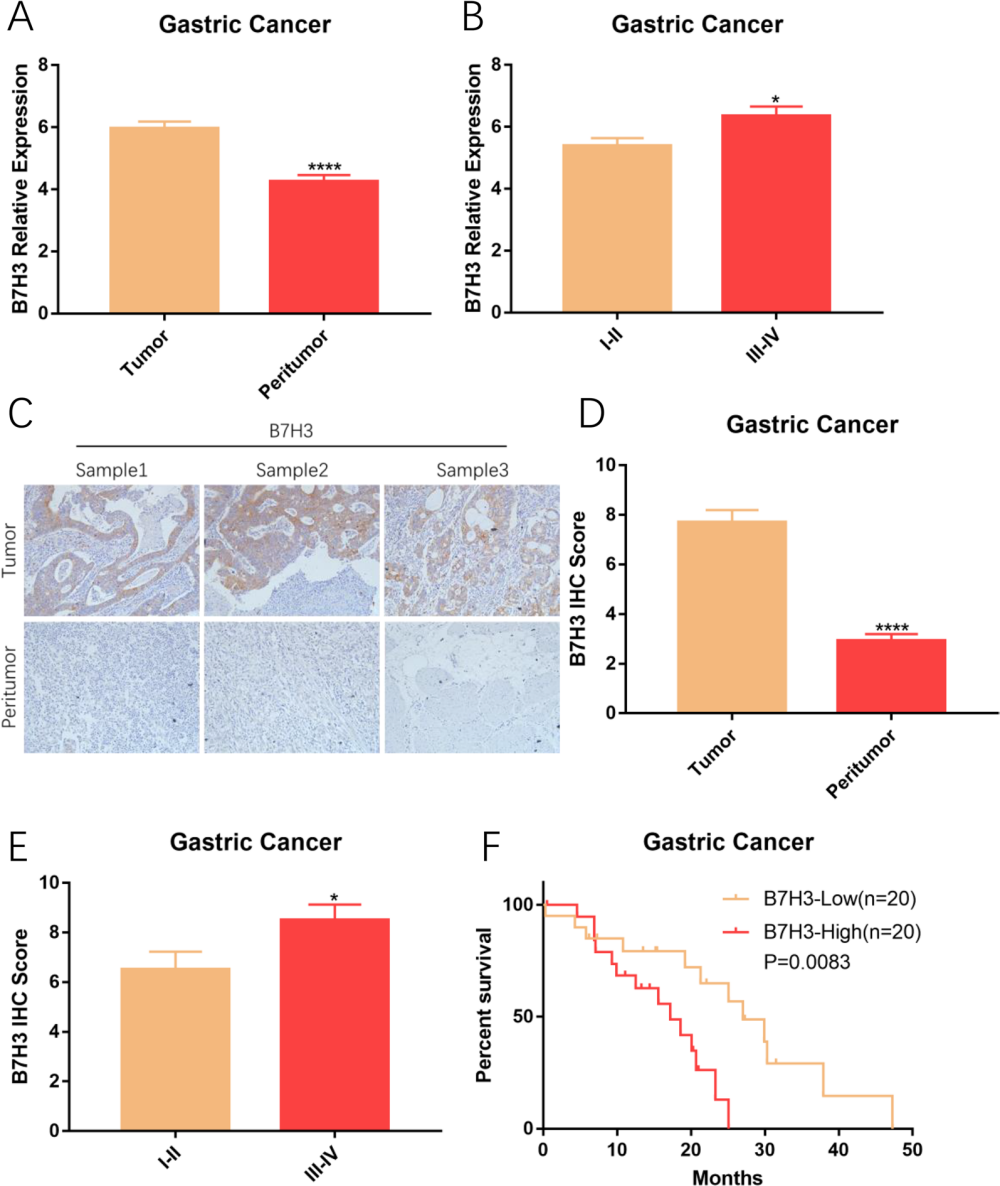
B7H3 is highly expressed on GC. **A** B7H3 was expressed at high levels in GC (****P < 0.0001). **B** Relationship between B7H3 and TNM stage (*P < 0.05). **C** Representative images from IHC analyses of B7H3 (×200). **D** IHC Score of B7H3 was high in GC (****P < 0.0001). **E** IHC Score of B7H3 was evaluated in samples from patients with different stages of GC (*P < 0.05). **F** Higher expression of B7H3 is associated with shorter survival in patients

To analyze the expression of B7H3 in GC, mRNA extracted from tumor and peritumor tissues was used for RT-PCR assay. A total of 80 samples, including 40 tumor tissues and 40 peritumor tissues, were collected from 40 patients with GC. Results of RT-PCR assay are shown that B7H3 is highly expressed on GC, furthermore, expression of B7H3 is significantly associated with the clinical stage of GC patients (Fig. 1A and B). We next characterized tumor B7H3 protein levels using immunohistochemistry (IHC) in specimens from GC patients. Detailed IHC score of B7H3 in GC patients are shown in Additional file 4: Table S3. Immunohistochemical findings are consistent with the result of RT-PCR assay (Fig. 1C–E). To appreciate if tumor B7H3 might influence patient survival, survival information of patients was collected and split into two groups according to the expression of B7H3. Survival analysis indicated the close correlation between B7H3 expression and the clinical survival time in patients with GC (Fig. 1F). These results all indicated that B7H3 is highly expressed on GC and correlated with disease advancement, it is suggested that B7H3 could serve as a potential therapeutic target for GC.

### Generation of anti-B7H3 CAR-T cells

**Fig. 2:**
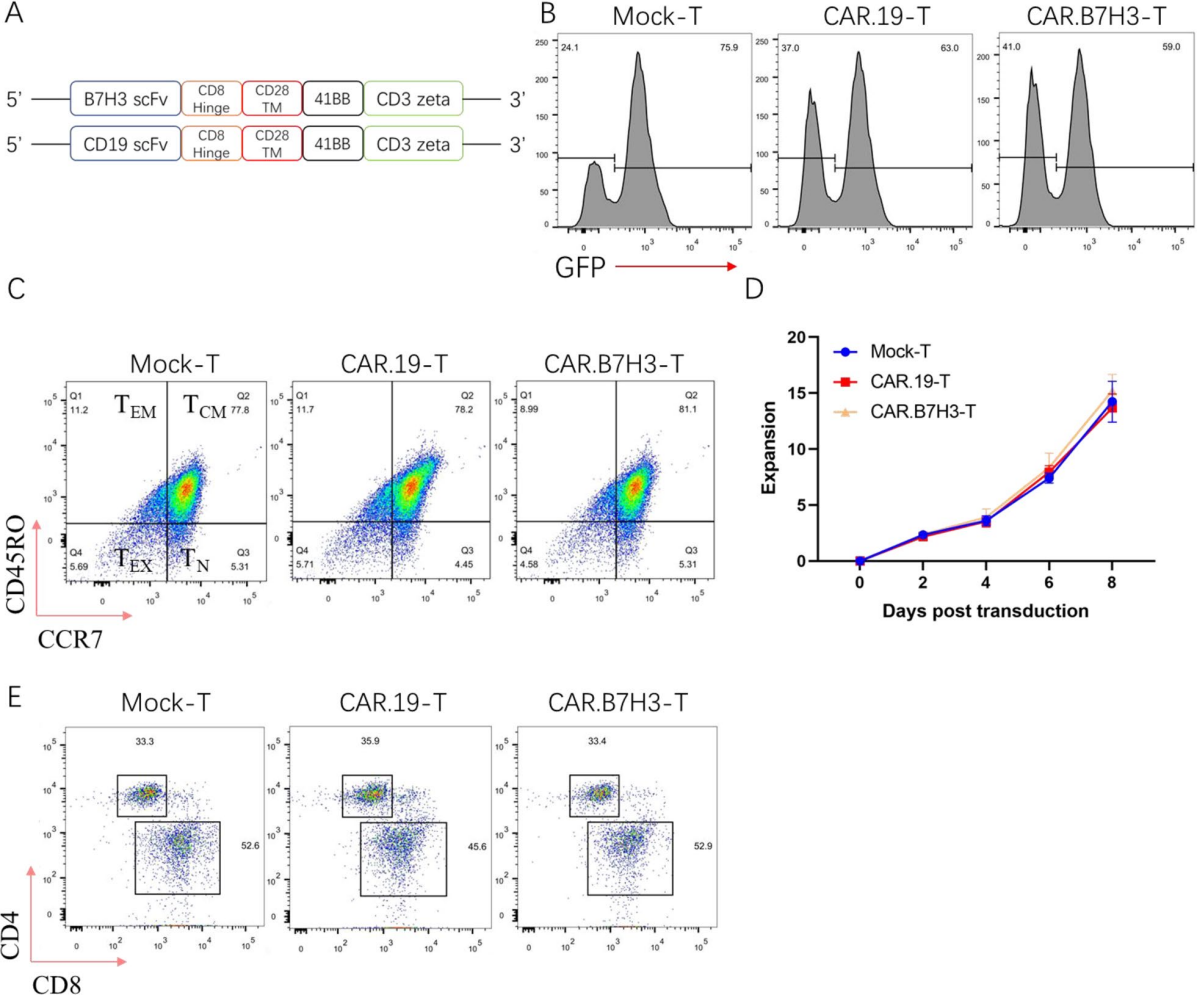
Generation of CAR-T cells. **A** Structures of CAR used. **B** Representative flow plot showing surface GFP expression on sorted CD3+ T cells. **C** T-cell differentiation after CAR transfected. **D** T-cell expanded after CAR transfected. **E** Flow cytometry analysis of T cells in three groups for CD4+ and CD8+ T cells ratios

Based on the highly expression of B7H3 in GC, CAR-T cells targeted B7H3 (CAR.B7H3-T) was constructed. In addition, CD19 directed CAR-T cells (CAR. CD19-T) was used as a control (Fig. 2A). Transfection efficiency and memory differentiation of CAR-T cells were identified on the seventh day. The GFP was used to detect the transfection efficiency and the transduction efficacy was comparable among the three groups of T cells (Fig. 2B), and lentiviral transfection did not affect T-cell differentiation in vitro (Fig. 2C). T cell proliferation is critical for an effective anti-tumor response, the result showed that the engineered T cells proliferated exponentially with minimally different kinetics (Fig. 2D). Subsets (CD4, CD8) of T cells were detected on the seventh day post-lentiviral infection by flow cytometry. Our results indicated there no differences about CD4+ T and CD8+ T cell ratios in the three groups. T cells in three groups showed a similar percentage of CD4+ and CD8+ T cells. The results showed that lentiviral infection did not affect ratios of CD4+ T and CD8+ T cells (Fig. 2E). All these results proved that successfully constructed CAR-T cells exhibited good viability and proliferation capacity. Thus, the CAR-T cells were used in the following studies.

### Anti‑B7H3 CAR‑T cells exhibit acutely cytotoxicity against GC cell lines

**Fig. 3:**
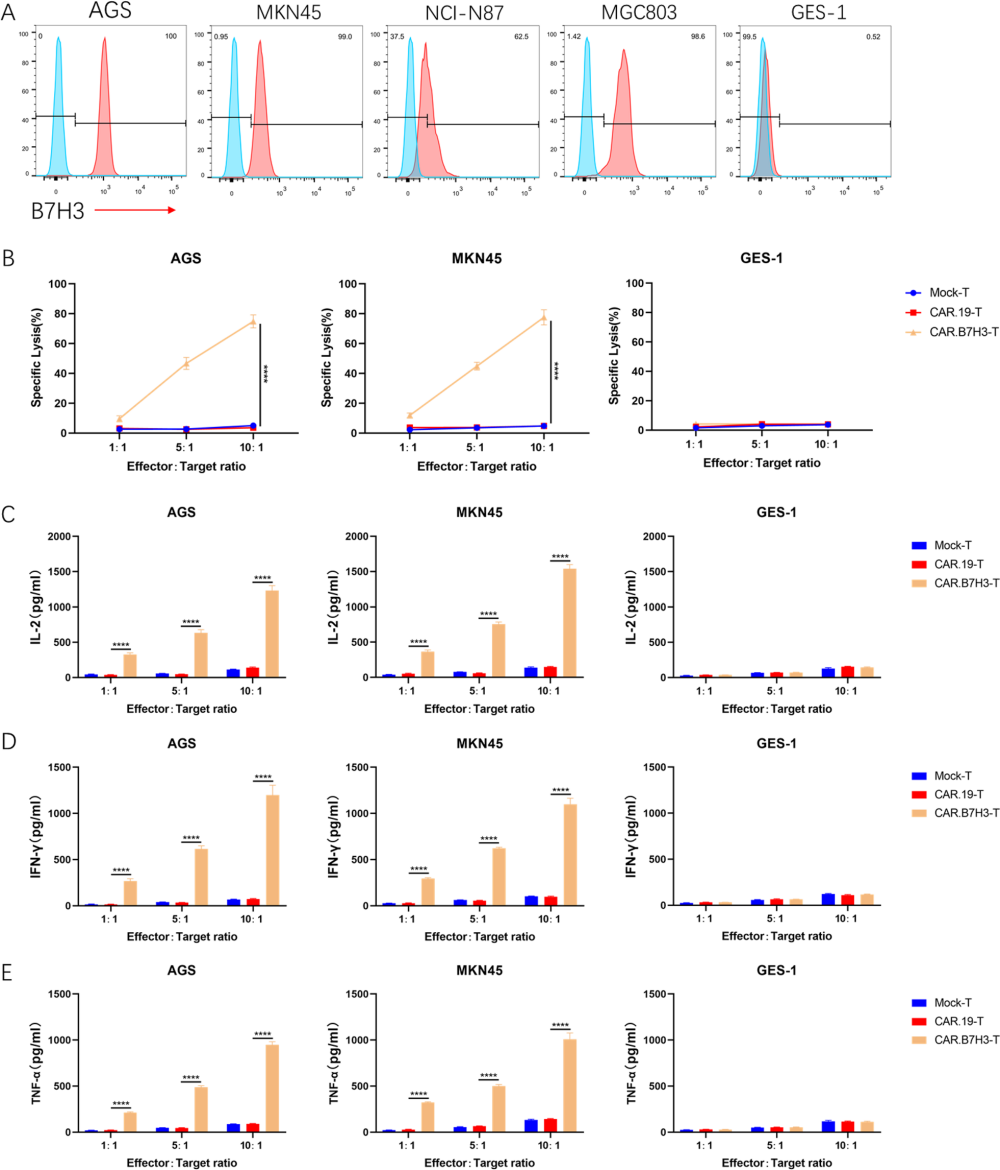
Antigen-dependent cytotoxicity of CAR-T cells. **A** B7H3 expression on GC cell lines. **B** Direct killing was performed using the LDH release assay (****P < 0.0001). **C**–**E** IL-2, IFN-γ and TNF-α production in co-cultures (****P < 0.0001)

To evaluate the clinical treatment potential of B7H3 specific CAR-T cells, the level of B7H3 on the cell surface of four GC cell lines (AGS, MKN45, NCI-N87and MGC803) and the normal gastric epithelial cell line GES-1was first detected by flow cytometry. Most GC cell lines exhibited high levels of B7H3 expression. However, GES-1 exhibited very faint CD276 staining (Fig. 3A). To verify whether CAR.B7H3-T cells had specific cytotoxic activity against B7H3 positive cell lines, CAR-T cells were then co-cultured with AGS, MKN45 and GES-1 cells. Compared with Mock-T and CAR.CD19-T cells, CAR.B7H3-T cells displayed robust cytotoxicity against AGS and MKN45 cells (Fig. 3B). In addition, the specificity was demonstrated by the absence of killing activity exhibited in Mock-T and CAR.CD19-T cells, and by the lack of cytotoxicity upon B7H3-negative GES-1 cells. CAR.B7H3-T cells were then tested for their cytokine secretion ability. IL-2 secretion by CAR.B7H3-T cells was significantly induced compared with that of Mock-T cells and CAR.CD19-T cells (Fig. 3C). Similar results were found for IFN-γ and TNF-α release (Fig. 3D, E). All these results proved that CAR.B7H3-T cells had specific cytotoxic activity against B7H3 positive cells. These observations suggested the clinical treatment potential of B7H3 specific CAR-T cells in GC.

### B7H3 expression positively correlates with the tumor stemness

**Fig. 4:**
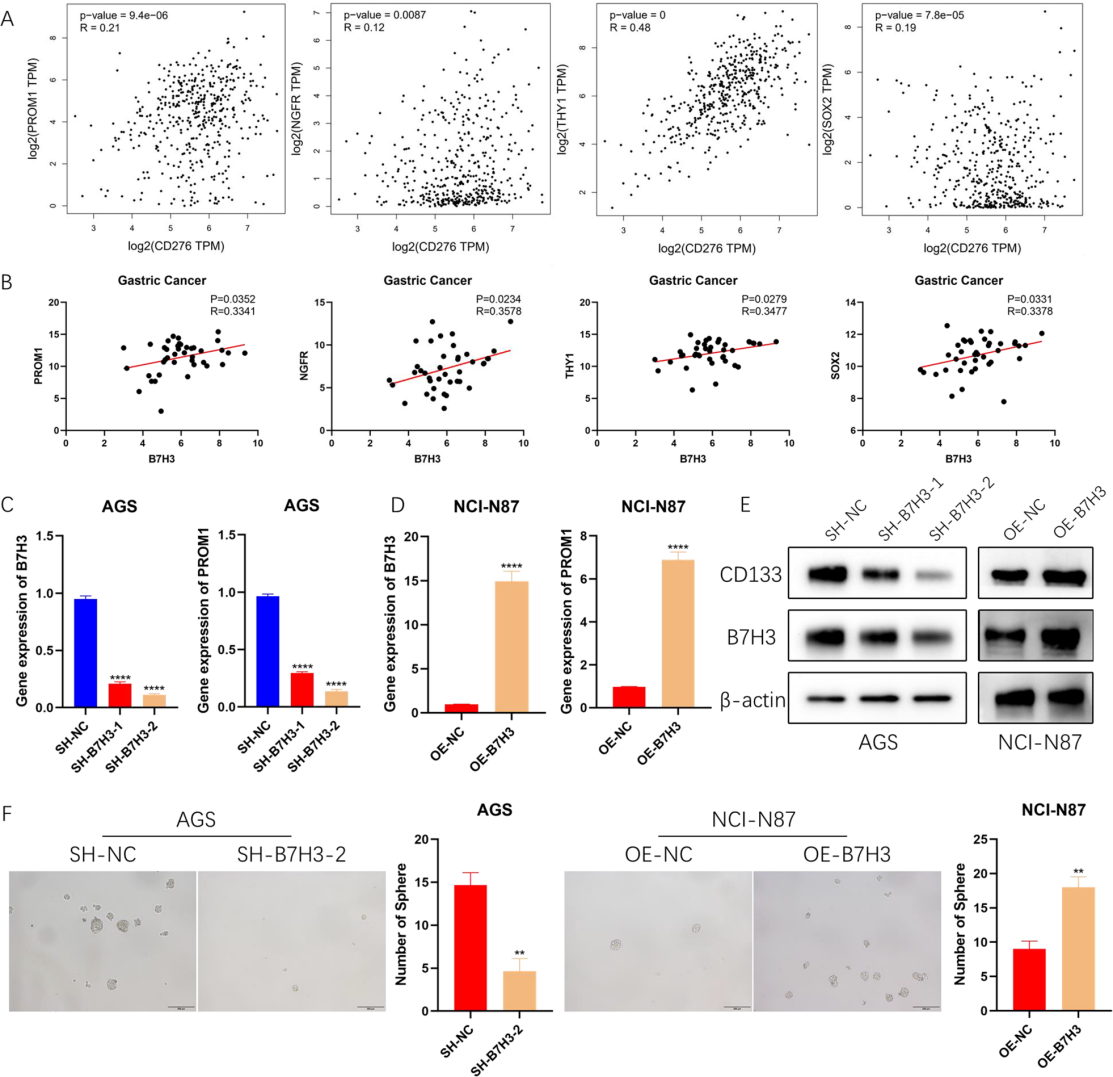
B7H3 expression positively correlates with the tumor stemness. **A** TCGA analysis showed that B7H3 significantly correlates cancer stem cell makers. **B** RT-PCR analysis showed that B7H3 significantly correlates cancer stem cell makers. **C** The expression of PROM1 was significantly decreased in AGS cells when B7H3 was knocked down (****P < 0.0001). **D** The expression of PROM1 was significantly up-regulated in NCI-N87 cells when B7H3 was overexpression (****P < 0.0001). **E** Protein levels of B7H3 and CD133 when the expression of B7H3 was changed. **F** The effect of B7H3 on sphere-forming ability of tumor cells (**P < 0.01)

Cancer stemness has been one of the most important potential mechanisms leading to tumor progression and recurrence. To elucidate whether B7H3 is associated with the stem cell-like properties of GC cells, expression of B7H3 and stemness markers in GC from TCGA dataset was analyzed. Correlation analysis showed that the positive correlation between B7H3 and stemness markers (*PROM1, NGFR, TYH1, SOX2*) was evident (Fig. 4A). Afterwards, this result was further confirmed by RT-PCR analysis in primary GC samples (Fig. 4B). To further verify the regulatory role of B7H3 on cancer cell stemness, B7H3 silencing plasmids and B7H3 overexpression plasmids were constructed, and then transfected into AGS and NCI-N87 cells. RT-PCR and WB were used to detected the change of B7H3 expression (Fig. 4C–E). B7H3 overexpression increased the expression of PROM1, and B7H3 silencing inhibited the expression of PROM1. The same trend was detected in the expression of NGFR, TYH1 and SOX2 (Additional file 1: Fig. S1A, B). Then, a sphere-forming assay indicated that depletion of B7H3 significantly suppressed stemness of AGS cells. In contrast, NCI-N87 cells with B7H3 overexpression exhibited more stemness (Fig. 4F). These data indicated that B7H3 can regulate stemness of GC cells.

### B7H3-directed CAR-T cells efficiently kill GC stem cells

**Fig. 5:**
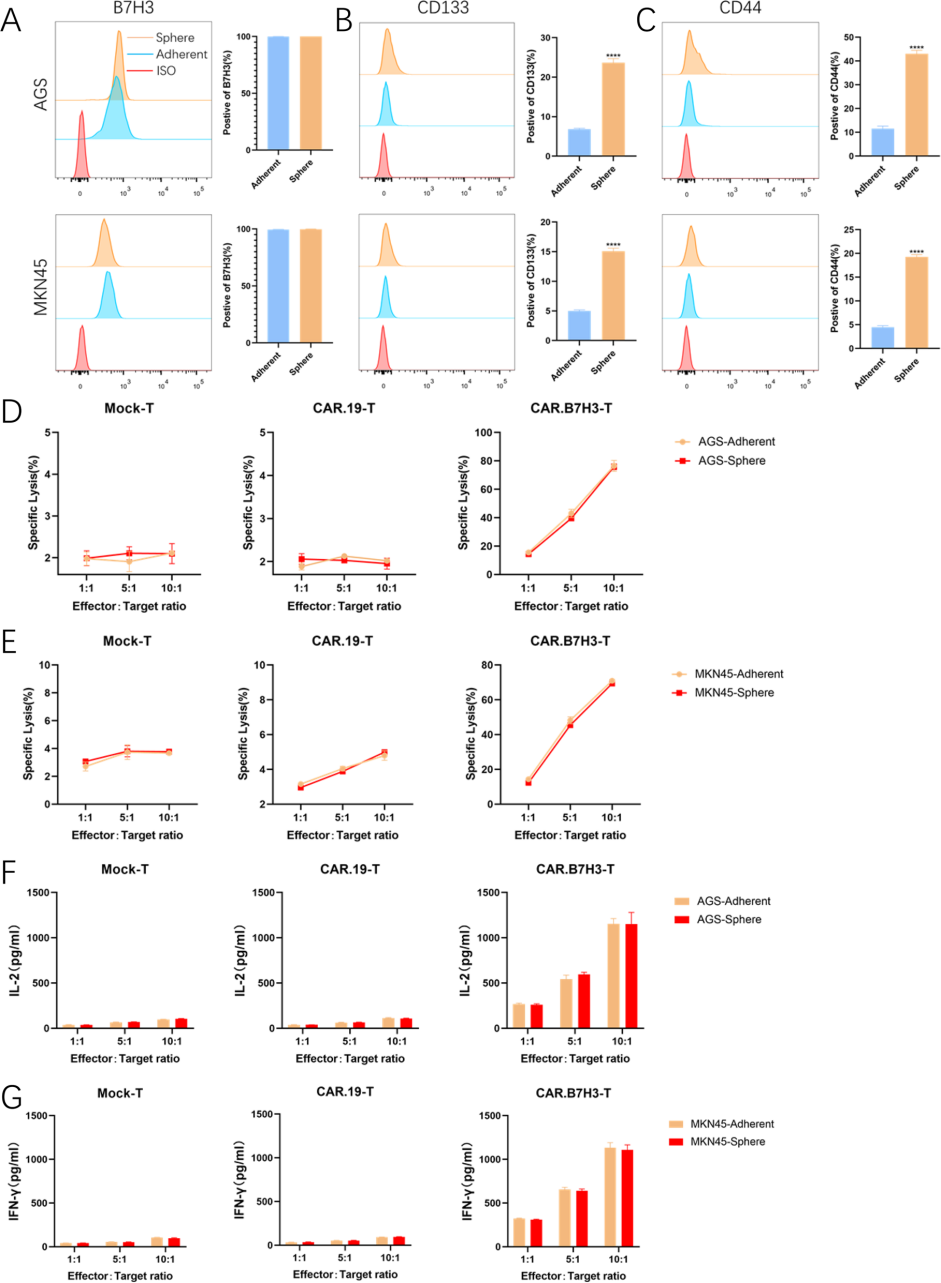
CAR.B7H3-T cells kill cancer stem cells. **A**–**C** Surface expressions of **A** B7H3, **B** CD133, and **C** CD276 were detected in tumor cells (adherent) and CSCs (sphere), respectively (****P < 0.0001). **D**, **E** Tumor cells (adherent) and CSCs (sphere) were co-culture with CAR.B7H3-T cells, and direct killing was performed using the LDH release assay. **F**, **G** ELISA of effector cytokines from CAR-T cells incubated with adherent or sphere tumor cells

Cancer stem cells (CSCs) are implicated in the development of treatment resistance in many malignancies. Hence, the capacity of CAR-T cells to kill CSCs is critically important for therapeutic success. To assess the therapeutic potential of CAR.B7H3-T cells, CSCs were enriched under sphere culture conditions. Compared with adherently cultured GC cells, CSC markers (CD133 and CD44) in AGS and MKN45 were up-regulated after sphere culture. However, expression of B7H3 did not change in these cell lines (Fig. 5A–C). To verify if CSCs could be eliminated as efficiently as tumor cells, Tumor cells were dissociated into single cells after sphere or adherent culture and then incubated with CAR-T cells. The results from the co-culture experiments proved that similar apoptosis levels were induced by CAR-T cells between sphere and adherent GC cells (Fig. 5D, E). Then, cytokine secretion was measured in the co-culture system. CAR.B7H3-T cells produced comparable amounts of cytokines upon GC cell or GC stem cells (Fig. 5F, G). These results proved that CAR.B7H3-T cells could efficiently kill GC stem cells.

### Anti-tumor effects of CAR.B7H3-T cells in vivo

**Fig. 6:**
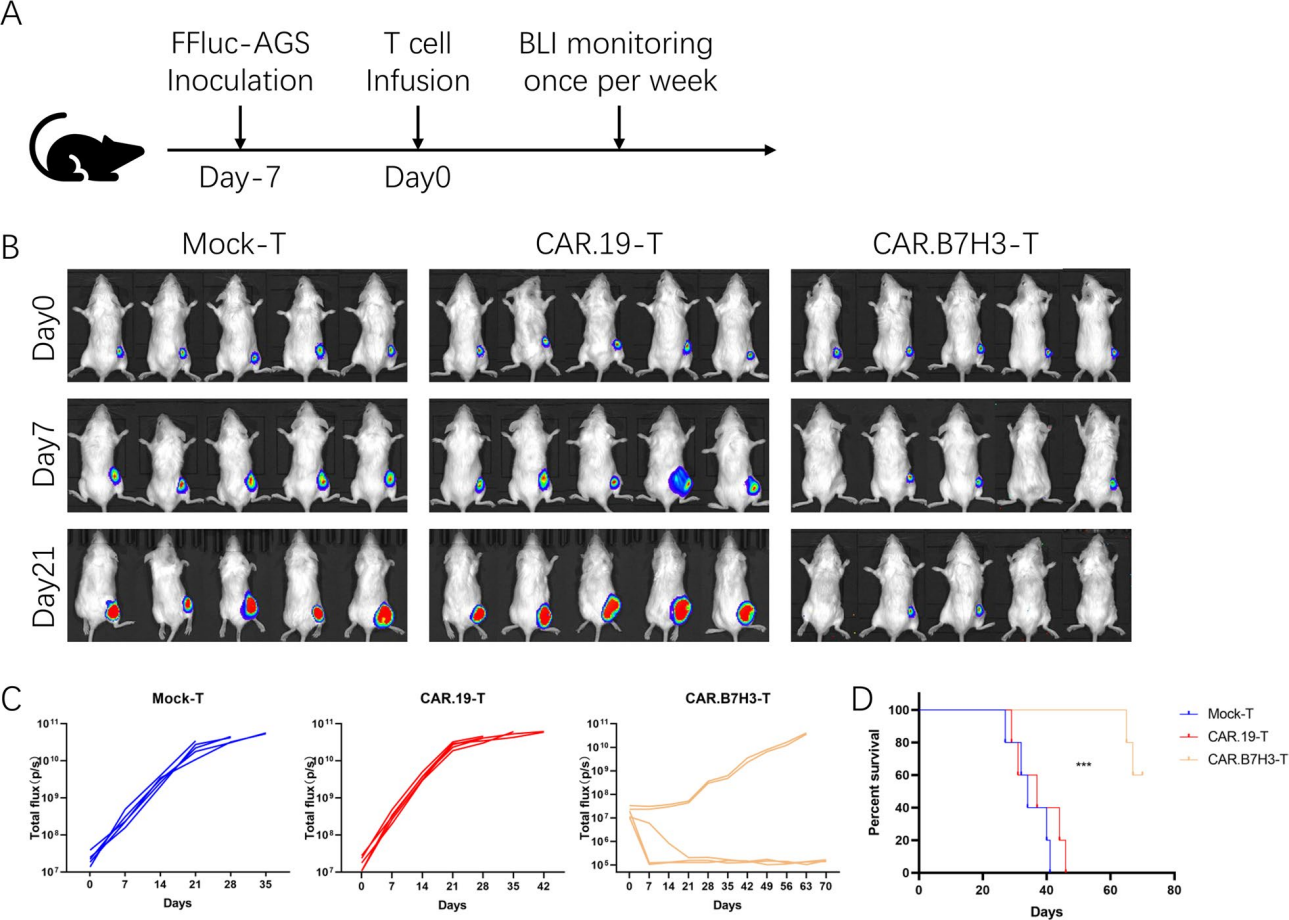
Anti-tumor effects of CAR.B7H3-T cells in vivo. **A** A schema of the animal experiment. **B** Tumor progression and distribution were evaluated by serial bioluminescence imaging. **C** Bioluminescence values of mice receiving different treatments are displayed. **D** The survival of the mice was monitored (***P < 0.001)

**Fig. 7:**
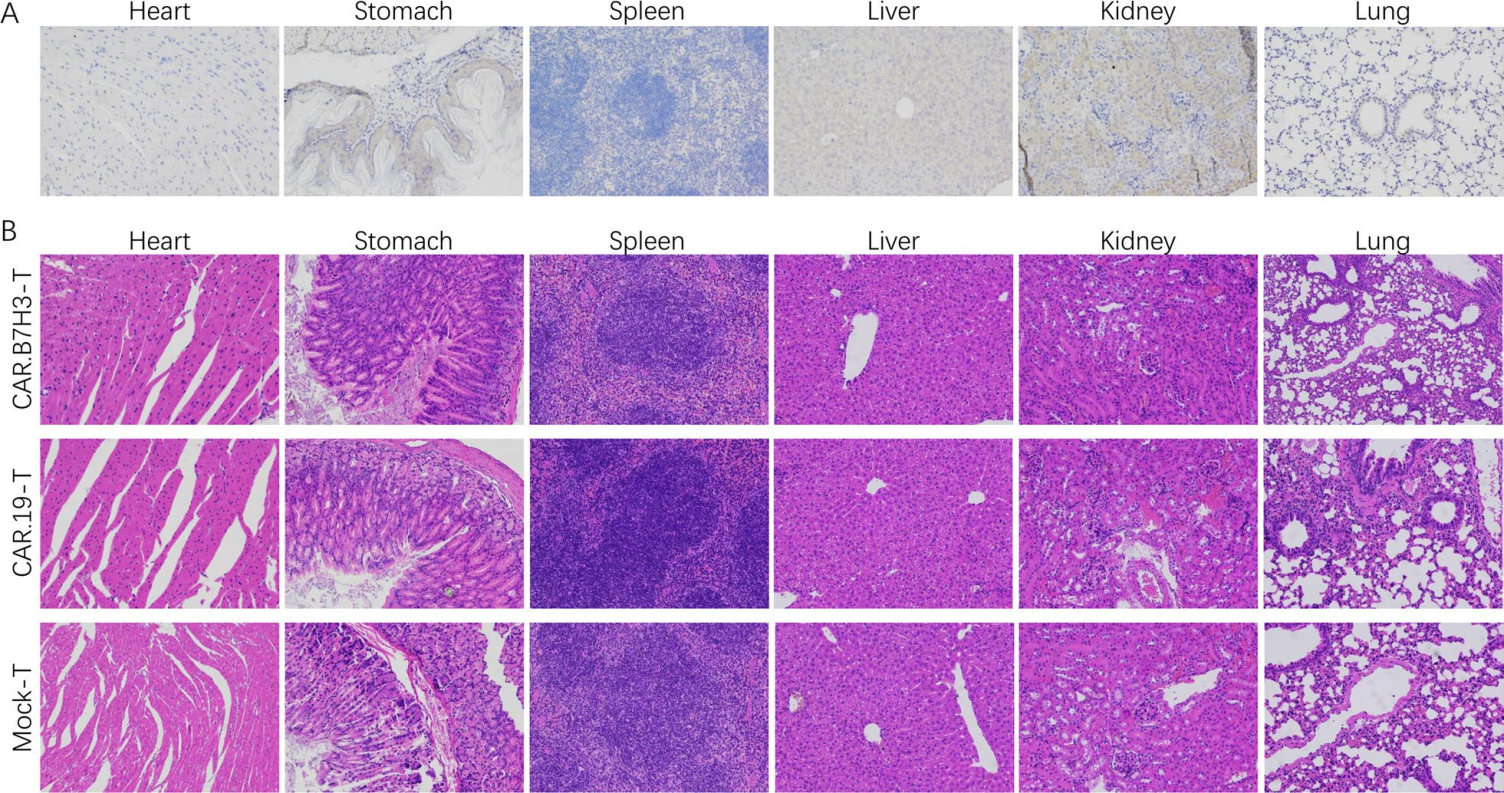
Safety evaluation of CAR.B7H3-T cells in GC xenotransplantation models. **A** IHC analysis of B7H3 expression in healthy tissues from vital organs in GC xenotransplantation models. **B** Pathological inspections of vital organs were performed to investigate pathological changes by H&E staining in GC xenotransplantation models

The anti-tumor activity of CAR.B7H3-T cells was preliminarily proven in vitro. Based on the above experimental results, we evaluated the anti-tumor activity of CAR.B7H3-T cells in vivo. FFluc-expressing AGS cells (3 × 10^5^, 100 μL) were used to establish tumors in the flank of NSG mice (n = 5). After 7 days, Mock-T, CAR.CD19-T and CAR.B7H3-T cells (1 × 10^6^, 100 μL) were injected intravenously. Subsequently, bioluminescence imaging system was used to observe the signal of tumor cells every week (Fig. 6A). By day 21, we observed substantially clearance of tumor cells in CAR.B7H3-T cells treatment group without recurrence whereas the tumor growth increased in the Mock-T cell group and CAR.CD19-T cell group (Fig. 6B, C). This result demonstrated that CAR.B7H3-T cells were potent in eliminating B7H3 positive tumor cells in NSG mice. Moreover, the contribution of CAR.B7H3-T cells to mouse survival was assessed. All of mice were died in the Mock-T cell group and CAR.CD19-T cell group by day 46, whereas 3/5 mice survived in CAR.B7H3-T cells treatment group during the experimental period (Fig. 6D). Taken together, these observations suggest that CAR.B7H3-T cells was effective in controlling tumor progression in vivo, and prolonged survival. To future evaluate the safety of B7H3-specific CAR-T cells, B7H3 expression in vital organs were performed by IHC staining in GC xenotransplantation models. IHC showed that the B7H3 antibody did not stain vital organs (Fig. 7A). Pathological inspections of vital organs were performed to investigate pathological changes by H&E staining in GC xenotransplantation models. Results showed that no obvious pathological changes were found in heart, liver, lung, stomach, spleen, and kidney sections in any of the treatment groups (Fig. 7B). In combination with the previous safety targeting GC effect of CAR.B7H3-T cells both in vivo and in vitro, we might draw a conclusion that CAR.B7H3-T cells represent an emerging immunotherapy for the treatment of GC.

## Discussion

Antigen is the key factor of antitumor efficacy and safety in CAR-T cell therapy [18]. High heterogeneity is an important characteristic of solid tumor and the leading cause of restricting CAR-T cell efficacy [19]. Therefore, identification of widely cancer-specific antigens is critical for CAR-T cell therapy. Again, novel targets are still urgently needed for enhancing the clinical efficacy of GC treatments.

As a transmembrane protein, B7H3 broadly expressed on many solid tumors but limited in healthy tissues, such as Ewing sarcoma, Wilms tumor, rhabdomyosarcoma, neuroblastoma as well as medulloblastoma [20]. Previous reports showed that 8H9S3.3 antibody (specific target B7H3) has already been used in the tumor therapy for more than 10 years [21]. This fully demonstrates the safety and effectiveness of target B7H3 in clinical application. In addition, B7H3 is also currently being explored as a target for CAR-T cell therapy and demonstrated safety and efficacy in mouse mode [14, 15, 17, 22, 23]. However, there are no reports on the expression of B7H3 in GC.

In our study, we examined the expression of B7H3 in GC tissues firstly. Our results showed that B7H3 expression was upregulated and exhibited extensive coverage of the tumor cells in GC. Meanwhile, B7H3-positive patients had a shorter survival than did B7H3-negative patients. These differences make B7H3 a good immunotherapeutic target. Then, we detected the antitumor efficacy of second-generation B7H3-targeting CAR T cells and demonstrated antigen-specific cytokine production and cytotoxicity in vitro. Subsequently, the positive correlation between B7H3 and CSCs was confirmed. This underscores the value of B7H3 for CAR-T cell therapy. Elimination of CSCs is essential for preventing tumor relapse, we further verified the killing effect of B7H3-spetific CAR-T cells to CSCs. The results proved that B7H3-specific CAR-T cells could efficiently kill CSCs. This suggests that B7H3-specific CAR-T cells therapy may offer a feasible immunotherapeutic strategy for patients with other treatment-resistant primary cancers with detectable B7H3 positive populations. Based on the results in vitro, we evaluated the anti-tumor activity of B7H3-specific CAR-T cells in vivo. These in vivo results demonstrated the therapeutic potential of B7H3-specific CAR-T cells for GC.

Our study highlights B7H3-specific CAR-T cells as a great potential therapeutic strategy for GC. Nevertheless, several limitations remain to be addressed. First, our investigations did not use an orthotopic tumor model of GC. Thus, the trafficking of CAR-T cells into malignant tissues cannot be unequivocally demonstrated. Second, the impact of the tumor microenvironment for CAR-T cells is not proven. This is also a key factor for CAR-T cell therapy. Third, the safety in clinical therapy is not clear. These questions represent important lines of our future work.

## Conclusions

In summary, we report on a B7H3-specific CAR-T cells that shows strong activity against GC. This CAR-T cells may provide a new therapeutic option for patients with advanced GC and should be carefully studied in early-phase clinical trials.

## Supplementary Information

**Additional file 1: Fig S1.** B7H3 modulates genes expression of NGFR, TYH1 and SOX2 in GC cells. (A) Expressions of NGFR, TYH1 and SOX2 were detected by RT-PCR when B7H3 was loss in AGS cells (***P < 0.001, ****P < 0.0001). (A) Expressions of NGFR, TYH1 and SOX2 were detected by RT-PCR when B7H3 was overexpression in NCI-N87 cells (***P < 0.001, ****P < 0.0001).

**Additional file 2: Table S1.** Primers used for RT-PCT.

**Additional file 3: Table S2.** Clinical characteristics in GC patients.

**Additional file 4: Table S3.** IHC score of B7H3 in GC patients.

## Data Availability

The datasets used and/or analyzed during the current study are available from the corresponding author on reasonable request.

## Abbreviations

GC: Gastric cancer
CAR: Chimeric antigen receptor
TCGA: The Cancer Genome Atlas
IHC: Immunohistochemistry
FFluc: Firefly luciferase
FN-γ: Interferon-γ
IL-2: Interleukin-2
TNF-α: Tumor necrosis factor-α
BLI: Bioluminescence imaging
ELISA: Enzyme-linked immunosorbent assay
CSC: Cancer stem cell
