# Supplementary figures and images for: Antitumor responses in gastric cancer by targeting B7H3 via chimeric antigen receptor T cells
Source: Cancer Cell Int. 2022 Jan 31;22:50. doi: 10.1186/s12935-022-02471-8 (PMC8802437; doi:10.1186/s12935-022-02471-8)

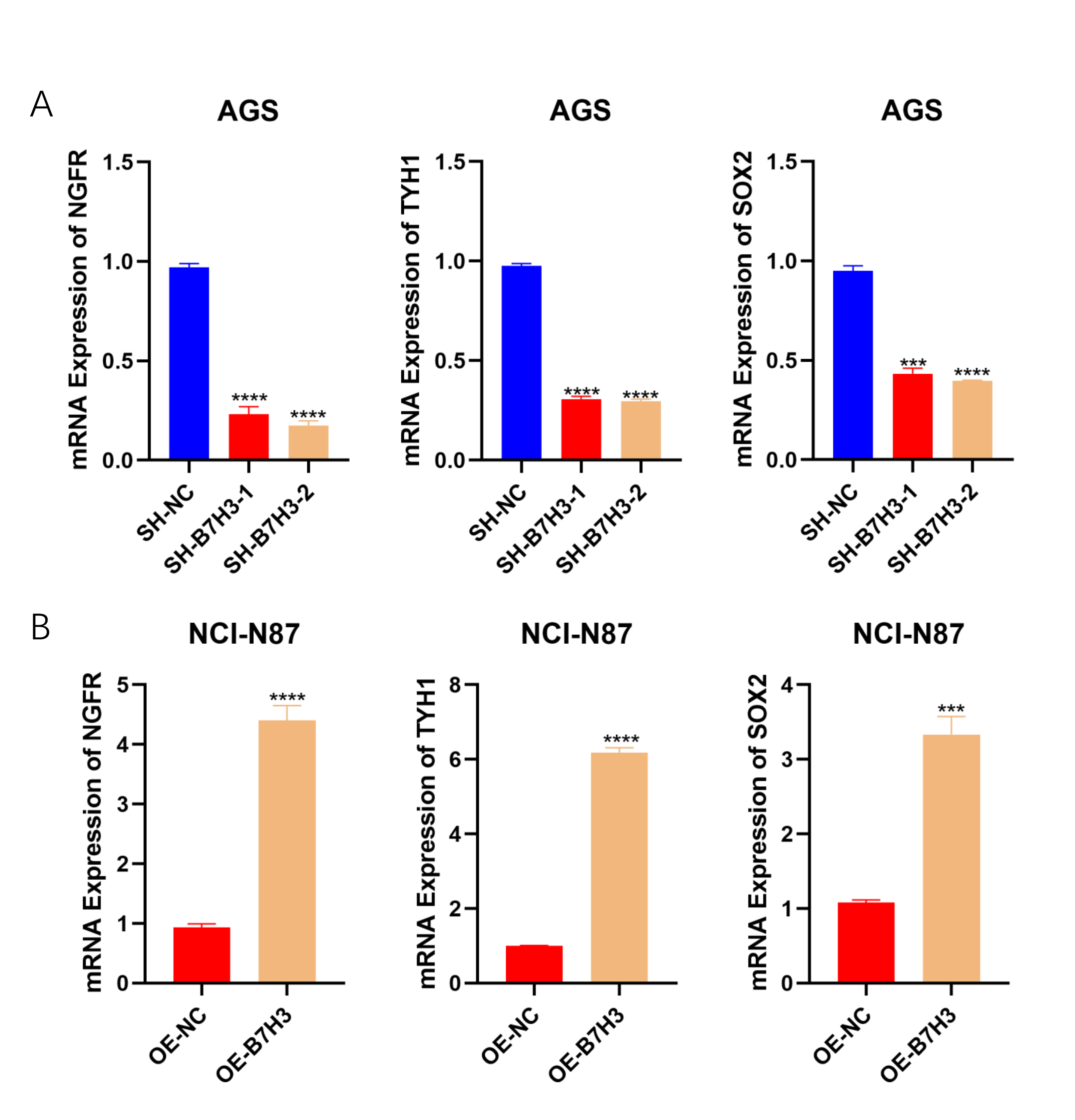

Supplement: Supplementary file 1 — Additional file 1: Fig S1. B7H3 modulates genes expression of NGFR, TYH1 and SOX2 in GC cells. (A) Expressions of NGFR, TYH1 and SOX2 were detected by RT-PCR when B7H3 was loss in AGS cells (***P < 0.001, ****P < 0.0001). (A) Expressions of NGFR, TYH1 and SOX2 were detected by RT-PCR when B7H3 was overexpression in NCI-N87 cells (***P < 0.001, ****P < 0.0001). [file 12935_2022_2471_MOESM1_ESM.tif]
