# Supplementary material for: Antitumor responses in gastric cancer by targeting B7H3 via chimeric antigen receptor T cells
Source: Cancer Cell Int. 2022 Jan 31;22:50. doi: 10.1186/s12935-022-02471-8 (PMC8802437; doi:10.1186/s12935-022-02471-8)
Supplement: Supplementary file 2 — Additional file 2: Table S1. Primers used for RT-PCT. [file 12935_2022_2471_MOESM2_ESM.docx]

**Table S1.** Primers used for qRT-PCT.

| Gene | Forward (5'-3') | Reverse (5'-3') |
| --- | --- | --- |
| B7H3 | CAAGGCAATGCATCCCTGAG | CTTCGAGTAGGGAGCGGC |
| GAPDH | TGACTTCAACAGCGACACCCA | CACCCTGTTGCTGTAGCCAAA |
| PROM1 | GGCCCAGTACAACACTACCAA | ATTCCGCCTCCTAGCACTGAA |
| NGFR  SOX2 | GCAGCCTATGCAGAATGGAGA  TCCCGTATGAAAGCATCGTGG | CCCCATAAGGATACGCAGACA  CCCATTTGGGTAGATCAGGTA |
| THY1 | ATCGCTCTCCTGCTAACAGTC | CTCGTACTGGATGGGTGAACT |
