# Supplementary material for: Antitumor responses in gastric cancer by targeting B7H3 via chimeric antigen receptor T cells
Source: Cancer Cell Int. 2022 Jan 31;22:50. doi: 10.1186/s12935-022-02471-8 (PMC8802437; doi:10.1186/s12935-022-02471-8)
Supplement: Supplementary file 3 — Additional file 3: Table S2. Clinical characteristics in GC patients. [file 12935_2022_2471_MOESM3_ESM.docx]

**Table S2.** Clinical characteristics in GC patients.

| Clinicopathological parameters | | Number of cases | | |
| --- | --- | --- | --- | --- |
| Sex |  | | |  |
| Male | | | 18 | |
| Female | | | 22 | |
| Age (years) | | |  | |
| <60 | | | 11 | |
| ≥60 | | | 29 | |
| TNM stage | | |  | |
| I-II | | | 16 | |
| III-IV | | | 24 | |
| Hp Infection | | |  | |
| Yes | | | 30 | |
| No | | | 10 | |
| Chemotherapy status | | |  | |
| Sensitive | | | 19 | |
| Resistant | | | 21 | |
| Surgical removal | | |  | |
| Yes | | | 40 | |
| No | | | 0 | |
