# Supplementary material for: Antitumor responses in gastric cancer by targeting B7H3 via chimeric antigen receptor T cells
Source: Cancer Cell Int. 2022 Jan 31;22:50. doi: 10.1186/s12935-022-02471-8 (PMC8802437; doi:10.1186/s12935-022-02471-8)
Supplement: Supplementary file 4 — Additional file 4: Table S3. IHC score of B7H3 in GC patients. [file 12935_2022_2471_MOESM4_ESM.docx]

**Table S3.** IHC score of B7H3 in GC patients.

| Patients | | IHC score of tumors  Intensity extent | | | | | | | | IHC score of peritumors  Intensity extent | | | | | | | |  |
| --- | --- | --- | --- | --- | --- | --- | --- | --- | --- | --- | --- | --- | --- | --- | --- | --- | --- | --- |
| TNM stage I-II |  | | |  | |  | | | | | |  | | | | |  |  |
| 1 | | | 3 | | | | | 4 | | | 0 | | | | 0 | | | |
| 2 | | | 2 | | | | | 4 | | | 1 | | | | 1 | | | |
| 3 | | | 3 | | | | | 3 | | | 1 | | | | 2 | | | |
| 4 | | | 2 | | | | | 3 | | | 1 | | | | 3 | | | |
| 5 | | | 2 | | | | | 3 | | | 2 | | | | 2 | | | |
| 6 | | | 2 | | | | | 4 | | | 2 | | | | 3 | | | |
| 7 | | | 3 | | | | | 3 | | | 1 | | | | 2 | | | |
| 8 | | | 3 | | | | | 4 | | | 1 | | | | 3 | | | |
| 9 | | | 2 | | | | | 2 | | | 1 | | | | 3 | | | |
| 10 | | | 2 | | | | | 2 | | | 1 | | | | 3 | | | |
| 11 | | | 3 | | | | | 2 | | | 2 | | | | 2 | | | |
| 12 | | | 2 | | | | | 2 | | | 2 | | | | 2 | | | |
| 13 | | | 2 | | | | | 2 | | | 2 | | | | 2 | | | |
| 14 | | | 2 | | | | | 3 | | | 2 | | | | 3 | | | |
| 15 | | | 1 | | | | | 3 | | | 1 | | | | 1 | | | |
| 16 | | | 3 | | | | | 1 | | | 0 | | | | 0 | | | |
| TNM stage III-IV | | |  | |  | | | |  | | | | |  | | | | |
| 1 | | | 2 | | | | | 4 | | | 1 | | | | 2 | | | |
| 2 | | | 2 | | | | | 3 | | | 1 | | | | 3 | | | |
| 3 | | | 2 | | | | | 3 | | | 2 | | | | 2 | | | |
| 4 | | | 3 | | | | | 3 | | | 2 | | | | 2 | | | |
| 5 | | | 3 | | | | | 2 | | | 2 | | | | 4 | | | |
| 6 | | | 2 | | | | 4 | | | | | | 1 | | | 2 | | |
| 7 | | | 3 | | | | 3 | | | | | | 1 | | | 2 | | |
| 8 | | | 3 | | | | 3 | | | | | | 0 | | | 0 | | |
| 9 | | | 3 | | | | 2 | | | | | | 1 | | | 3 | | |
| 10 | | | 3 | | | | 4 | | | | | | 1 | | | 3 | | |
| 11 | | | 3 | | | | 4 | | | | | | 2 | | | 2 | | |
| 12 | | | 2 | | | | 4 | | | | | | 2 | | | 2 | | |
| 13 | | | 2 | | | | 4 | | | | | | 2 | | | 3 | | |
| 14 | | | 3 | | | | 3 | | | | | | 1 | | | 3 | | |
| 15 | | | 3 | | | | 3 | | | | | | 2 | | | 2 | | |
| 16 | | | 1 | | | | 2 | | | | | | 1 | | | 2 | | |
| 17 | | | 2 | | | | 4 | | | | | | 1 | | | 2 | | |
| 18 | | | 3 | | | | 2 | | | | | | 1 | | | 1 | | |
| 19 | | | 3 | | | | 2 | | | | | | 1 | | | 1 | | |
| 20 | | | 2 | | | | 4 | | | | | | 1 | | | 2 | | |
| 21 | | | 2 | | | | 4 | | | | | | 1 | | | 3 | | |
| 22 | | | 3 | | | | 4 | | | | | | 1 | | | 2 | | |
| 23 | | | 3 | | | | 4 | | | | | | 2 | | | 2 | | |
| 24 | | | 3 | | | | 3 | | | | | | 1 | | | 2 | | |

TNM=tumor node metastasis. Intensity (0 = none, 1 = low, 2 = moderate, and 3 = high). Extent (0% = 0, 1–24% = 1, 25–49% = 2, 50–74% = 3, and 75–100% = 4).
